# Metropolitan-level ethnic residential segregation, racial identity, and body mass index among U.S. Hispanic adults: a multilevel cross-sectional study

**DOI:** 10.1186/1471-2458-14-283

**Published:** 2014-03-27

**Authors:** Kiarri N Kershaw, Sandra S Albrecht

**Affiliations:** 1 Department of Preventive Medicine, Northwestern University Feinberg School of Medicine, 680 N Lake Shore, Suite 1400, Chicago, IL, USA; 2 Carolina Population Center, University of North Carolina at Chapel Hil, 123 W Franklin St, Chapel Hill, NC, USA

**Keywords:** Hispanics, Residential segregation, Body mass index, Race, Health disparities

## Abstract

**Background:**

The few studies that have examined whether metropolitan-level ethnic residential segregation is associated with obesity among Hispanics are mixed. The segmented assimilation theory, which suggests patterns of integration for immigrant groups varies by social factors, may provide an explanation for these mixed findings. In this study we examined whether one social factor, racial identity, modified the association between ethnic residential segregation and body mass index (BMI) among Hispanics.

**Methods:**

We used data on 22,901 male and 37,335 non-pregnant female Hispanic adult participants of the 2003–2008 U.S. Behavioral Risk Factor Surveillance System living in 227 metropolitan or micropolitan areas (MMSAs). Participants self-identified as White, Black, and ‘some other race’. BMI was calculated using self-reported height and weight; the Hispanic isolation index was used to measure Hispanic residential segregation. Using multi-level linear regression models, we examined the association of Hispanic residential segregation with BMI, and we investigated whether this relationship varied by race.

**Results:**

Among men, Hispanic segregation was unassociated with BMI after adjusting for age, race, MMSA-level poverty, and MMSA-level population size; there was no variation in this relationship by race. Among women, significant associations between Hispanic segregation and BMI in models adjusted for demographics and MMSA-level confounders became attenuated with further adjustment for education and language of exam. However, there was statistically significant variation by race (*P*_interaction_ = 0.03 and 0.09 for Hispanic Blacks and Hispanics who identified as some other race, respectively, vs. Hispanic Whites). Specifically, higher segregation was associated with higher mean BMI among Hispanic Whites, but it was associated with lower mean BMI among Hispanic Blacks. Segregation was unassociated with BMI among Hispanic women identifying as some other race.

**Conclusions:**

This heterogeneity highlights the persistent influence of race on structural processes that can have downstream consequences on health. As Hispanics grow as a proportion of the U.S. population, especially across urban centers, understanding the health consequences of residence in segregated areas, and whether or not these impacts vary across different groups, will be important for the design of more comprehensive solutions to prevent adverse health outcomes.

## Background

Hispanics are the fastest growing minority group in the U.S., constituting over 16% of the total population, and accounting for more than half of the nation’s population growth in the last decade [1]. Similar to non-Hispanic Blacks, Hispanics carry a disproportionate burden of obesity relative to non-Hispanic Whites. Continuation of this patterning will have implications for future chronic disease burden in the U.S. Thus, a better understanding of the factors that contribute to obesity among Hispanics will help to better target public health prevention efforts.

A growing body of research suggests that contextual factors may influence obesity and contribute to racial and ethnic disparities [2-6]. Metropolitan-level racial or ethnic residential segregation, a process that sorts individuals into different neighborhoods by race or ethnicity, has been implicated as a fundamental cause of health disparities [7]. Residential location determines the availability and quality of economic and social resources including schools, safety, recreational amenities, and public transportation [7,8]. As a result, living in racially or ethnically segregated neighborhoods may also influence social, economic, and health outcomes [9].

Most studies of metropolitan-level racial segregation have focused on non-Hispanic Black populations and have shown that higher levels of segregation are associated with poorer health, including obesity, hypertension, and worse self-rated health [10-13]. However, it is unclear whether metropolitan-level residential segregation has the same implications for health among Hispanics. On the one hand, greater residential concentration of Hispanics is thought to be associated with higher area poverty, higher crime, and limited access to health-promoting resources [14,15]. On the other hand, it has also been associated with better social support and networks, and a greater availability of healthier food options [14,16-18]. Results from the few published empirical studies of metropolitan-level segregation and obesity among Hispanics are inconsistent. One study of metropolitan-level segregation and obesity in Mexican-Americans found higher segregation was associated with lower obesity among women; however there was no association among men [19]. In contrast, another study among Hispanics showed that higher segregation was associated with higher obesity; results were not presented separately for men and women [20].

These findings suggest there may be multiple, yet divergent pathways linking segregation to health. Given the heterogeneity of the Hispanic population, these mixed findings may also reflect variation in the impact of segregation across groups. For example, there is evidence that associations between segregation and health differ between men and women [19], and between immigrants and those born in the U.S. [21]. Another factor that may be a significant source of variation in this relationship is race. Although routinely examined under a single racial/ethnic classification, Hispanics represent a diverse continuum on the racial spectrum from white to black. A growing proportion, approximately 42% according to the 2000 U.S. Census, also identify as ‘some other race’ [22]. One reason for this is that many Hispanics do not identify with existing race options and instead, choose the ‘some other race’ category because it better reflects a racialized Hispanic identity that aligns more closely with their Latin American mestizo heritage [23,24].

Several studies have shown that socially defined race among Hispanics influences health [25]. For example, a study of blood pressure in a community-based sample of Puerto Ricans found that darker skin color defined by cultural consensus was associated with higher blood pressure, particularly among higher socioeconomic status individuals [26]. However, they found no association between skin pigmentation measured by a reflectometer and blood pressure. In addition, a U.S. study comparing associations of self-identified and socially assigned race with self-rated health found that among participants who self-identified as Hispanic, those who were usually classified by other people as White had better self-rated health than those who were usually classified by other people as Hispanic [27]. These studies highlight the importance of socially constructed race as a determinant of health among Hispanics.

Residence in racially or ethnically segregated areas has historically been the first step in the assimilation process for race or ethnic groups who immigrate to the U.S., including Hispanics [28]. There are three competing theories relating to how residential patterns among recent immigrant groups change over time [29]. Spatial assimilation theory states that immigrants become integrated with the majority group over time as they adopt mainstream cultures and attitudes and as they rise in socioeconomic status [30]. Place stratification theory posits that immigrants will remain segregated from the majority group across generations due to factors like discrimination by the majority group [31]. The segmented assimilation theory suggests patterns of integration will vary due to social factors such as race [18].

Although race has long played a role in shaping residential patterns in the U.S. among non-Hispanics, it has been shown to do so among Hispanics as well. Data from the 2000 Census indicates that Hispanic Whites are less likely to be segregated from non-Hispanic Whites than Hispanic Blacks [29]. Moreover, there is evidence that Hispanic Blacks are just as segregated from non-Hispanic Whites as non-Hispanic Blacks are [23]. These findings suggest that race may be a salient factor in determining where Hispanics live, especially as they move away from ethnic enclaves and assimilate to the U.S. mainstream. If the residential options outside of ethnic enclaves differ for Hispanic Blacks and Whites, then the health implications of living in an ethnic enclave could be expected to differ by race.

Using data from a large national sample of Hispanic men and women in the Behavioral Risk Factor Surveillance System (BRFSS), our study builds on existing research by examining the inter-relationships among residential segregation, racial identity, and body mass index (BMI). We investigated 1) whether Hispanic residential segregation is associated with differences in mean BMI and 2) whether the association between segregation and BMI varies by race. Only two other studies to our knowledge have assessed the association between metropolitan-level residential segregation and weight among Hispanics [19,20], and none have examined differences in this relationship by race. Consistent with the segmented assimilation theory, we hypothesized that associations of segregation with BMI would vary by race. As Hispanics grow as a proportion of the U.S. population, especially across urban centers, it will be important to understand the health consequences of residence in segregated areas, and whether or not these impacts vary across different groups.

## Methods

### Data source and study sample

This study used data from the 2003–2008 Behavioral Risk Factor Surveillance System (BRFSS). BRFSS is a cross-sectional, nationally representative random-digit-dial telephone survey administered annually by the Centers for Disease Control and Prevention [32]. The BRFSS survey was approved by the Institutional Review Boards of the Centers for Disease Control and Prevention and all participating states’ Departments of Health. We restricted analyses to Hispanics aged 25 years and older living in U.S. metropolitan or micropolitan statistical areas (MMSAs) that self-identified as White, Black, or ‘some other race.’ This is consistent with the primary race classifications selected by the majority of U.S. Hispanics in the 2000 U.S. Census [22]. Data were pooled across 6 years (2003–2008) of the BRFSS to ensure sufficient sample size for Hispanic Blacks.

### Measures

#### Outcome

BMI (weight in kilograms/height in meters squared) was calculated using self-reported height and weight, and was modeled continuously.

#### Hispanic residential segregation

Massey and Denton conceptualized five geographic dimensions of racial/ethnic residential segregation: evenness, exposure, clustering, centralization, and concentration [33]. All are empirically correlated, but each is thought to represent distinct aspects of residential segregation. In this study the Hispanic isolation index, a measure of the exposure dimension, was used. The Hispanic isolation index estimates the extent to which Hispanics are only exposed to other Hispanics (of any race). The Hispanic isolation index was created using 2005–2009 American Community Survey data [34]. Census tracts were used as proxies for neighborhoods in these analyses. The isolation index is a commonly used measure with a well-documented theoretical pathway through which it is believed to influence health outcomes [33]. Specifically, it is hypothesized to lead to health disparities by concentrating poverty among minorities and leaving them more vulnerable to the adverse health outcomes associated with living in disadvantaged neighborhoods [35]. The isolation index represents the average neighborhood % Hispanic in which the average Hispanic lives within a given metropolitan/micropolitan area. It is represented mathematically as follows [33]:

$$\mathrm{Isolation}\;\mathrm{index}={\;}_{x}{P^{*}}_{x}=\sum_{i=1}^{n}\left[\frac{x_{i}}{X}\right]\left[\frac{x_{i}}{t_{i}}\right]$$

Where *x*_*i*_ is the number of Hispanics in census tract *i*, *t*_*i*_ is the total population in tract *i* and *X* is the number of Hispanics in the metropolitan area. This proportion is then summed across all *n* census tracts in the MMSA. MMSAs are geographic entities consisting of urban areas and surrounding counties that are tied economically or socially to the urban core. The isolation index was only computed for MMSAs that have at least 1000 Hispanics, since segregation indices for smaller minority populations are less reliable [29].

#### Covariates

Race was categorized as Black, some other race, and White. Age was modeled continuously, and a squared term was included to account for its non-linear relationship with BMI. Education and acculturation were adjusted for as individual-level factors that might account for associations of segregation with BMI. These factors could be confounders, but given that segregation is hypothesized to contribute to health disparities by influencing opportunities for socioeconomic mobility (e.g., through poor quality schools), these may also be mediators [7]. Education was categorized as less than high school completed, high school diploma or equivalent, and more than high school. Nativity (foreign-born vs. U.S.-born) was not available in the BRFSS data. Therefore, the language in which the interview was administered, non-English versus English, was included, instead, as a proxy for acculturation, a process recognized as an important determinant of health among Hispanics [36]. The MMSA population size and the percentage of the MMSA population living below the U.S. Census Bureau-defined poverty threshold [37] were also adjusted for in these analyses as confounders.

### Analysis

All results were gender-stratified based on previous research showing differences in relationships of adversity and stress with eating behaviors and weight between men and women [38,39]. Of the 24,722 male and 42,577 non-pregnant female self-identified Hispanic Black, White, or some other race BRFSS participants aged 25 and older living in MMSAs with 1000 or more Hispanics, 1406 men and 4695 women were excluded for missing data on height or weight. An additional 415 men and 547 women were excluded for missing data on education or language of exam, yielding a sample of 22,901 men (842 Black, 11,982 White, and 10,077 some other race) and 37,335 women (1,421 Black, 19,978 White, and 15,936 some other race). Hispanic White men were less likely to have missing data than Hispanic Black or some other race men, while Hispanic women who identified as some other race were more likely to have missing data than Hispanic Black or White women.

Means with standard errors and frequencies were calculated for all continuous and categorical covariates by race, accounting for unequal selection probabilities. Continuous covariates were compared using analysis of variance and categorical variables were compared using the χ^2^ statistic. Descriptive analyses also examined the distribution of Hispanic Blacks, Hispanic Whites, and Hispanics who identified as some other race across categories (based on quartiles) of MMSA-level Hispanic isolation index score, poverty, and population. We also examined the distribution of non-Hispanic Black and White isolation index scores among Hispanics living in the lowest quartile of the Hispanic isolation index score. This was done to evaluate whether there was evidence of segmented assimilation patterns among Hispanics living outside ethnic enclaves by race.

Multilevel linear regression was used to estimate associations between residential segregation, race, and BMI. A series of two-level random intercept models (with a random intercept for each MMSA) were fitted using the 22,901 male and 37,335 female study participants nested within 227 MMSAs. There was an average of 100.9 men and 164.5 women per MMSA. Since there was no evidence of a non-linear relationship between the Hispanic isolation index and BMI, the index was modeled continuously. Estimates of the association between the isolation index and BMI correspond to mean differences in BMI per 1-standard deviation (SD) unit increase in the isolation index score. The first model was adjusted for age, age^2^, race, the Hispanic isolation index, and MMSA-level confounders. Subsequent models were additionally adjusted for the language of interview and education. To evaluate heterogeneity in this association by race, an interaction between race and the Hispanic isolation index was tested.

Individual-level sampling weights were incorporated into the multilevel models to account for the unequal selection probabilities of the participants. These weights were scaled so that the new weights summed to the level-2 (MMSA) cluster sample size [40]. Descriptive analyses were conducted using SAS survey procedures (SAS Institute, Cary, NC) and multilevel analyses were conducted using Mplus 6 (Muthén & Muthén, Los Angeles, CA).

## Results

Table 1 shows sample characteristics by sex and race. Among men, mean BMI for Hispanic Blacks and Whites was the same, (28.1 kg/m^2^) and among Hispanics who identified as some other race, it was slightly higher (28.3 kg/m^2^). Hispanics who identified as some other race were younger and had less education than Hispanic Whites. There were also similar age and educational differences between Hispanic Black and White men, but these were not statistically significant. Among women, mean BMI was significantly higher among Hispanic Blacks (28.7 kg/m^2^) and Hispanics who identified as some other race (28.0 kg/m^2^) compared to Hispanic Whites (27.6 kg/m^2^). Hispanic Black and some other race women were also younger, and a smaller percentage interviewed in a non-English language. However, there were no race differences in educational attainment.

**Table 1:** **Sample characteristics of Hispanics in the 2003**–**2008 Behavioral Risk Factor Surveillance System** (**BRFSS**) **by sex and race**

	**Men**			**Women**		
	**Hispanic blacks (n = 842)**	**Hispanics who identified as some other race (n = 10,077)**	**Hispanic whites (n = 11,982)**	**Hispanic blacks (n = 1421)**	**Hispanics who identified as some other race (n = 15,936)**	**Hispanic whites (n = 19,978)**
Body mass index, kg/m^2^ (SE)	28.1 (0.3)	28.3 (0.1)*	28.1 (0.1)	28.7 (0.4)*	28.0 (0.1)*	27.6 (0.1)
Age, years (SE)	41.2 (0.9)	40.4 (0.2)*	42.7 (0.2)	43.2 (0.8)*	42.0 (0.2)*	45.2 (0.2)
Education, %						
Less than high school	38.1	35.9*	31.8	31.6	31.2	31.7
High school	27.9	27.3	25.7	27.7	28.1	26.7
More than high school	33.9	36.8	42.6	40.7	40.7	41.6
Non-English questionnaire, %	41.2	43.9	43.3	36.6	39.2*	41.8

*Abbreviation:SE* = standard error.

*P < .05 for comparisons with Hispanic Whites.

Table 2 depicts the distribution of Hispanic study participants by race and quartiles of MMSA-level Hispanic isolation index score, poverty, and population size. Since distributions were similar for men and women, they were combined in this table. Hispanic Blacks, Whites, and those who identified as some other race were represented in all quartiles of the MMSA-level characteristics. A smaller percentage of Hispanic Black participants were living in areas of high Hispanic segregation areas (8.4% of all Hispanic Blacks) compared to low segregation areas (36.0%); and a smaller percentage resided in small MMSAs compared to large MMSAs (15.4% and 37.4%, respectively). Hispanic Whites and those who identified as some other race were evenly distributed across the quartiles for each characteristic.

**Table 2:** **Distribution** (**shown as percentage**) **of MMSA**-**level characteristics by race among Hispanics in the 2003**–**2008 Behavioral Risk Factor Surveillance System** (**BRFSS**)

**Quartile of MMSA-level characteristic**	**Hispanic blacks (n** **=** **2,263)**	**Hispanics who identified as some other race (n** **=** **26,013)**	**Hispanic whites (n** **=** **31,960)**
Hispanic isolation index			
Q1 (<0.24)	36.0	25.5	26.8
Q2 (0.24 – 0.369)	23.5	25.5	18.7
Q3 (0.37 – 0.539)	32.0	26.0	24.0
Q4 (≥ 0.54)	8.4	23.0	30.5
Mean poverty^a^			
Q1 (< 10.7%)	30.5	26.1	23.8
Q2 (10.7% – 13.0%)	29.6	23.3	25.9
Q3 (13.1% – 15.3%)	15.9	21.6	25.8
Q4 (≥ 15.4%)	24.0	29.0	24.5
Population size			
Q1 (< 695,157)	15.4	24.8	24.2
Q2 (695,157 – 1,480,259)	21.7	27.7	23.4
Q3 (1,480,259 – 2,669,986)	25.5	26.8	24.3
Q4 (≥ 2,669,986)	37.4	20.7	28.2

*Abbreviations:MMSA* = metropolitan or micropolitan statistical area; *Q* = quartile.

^a^Percentage of the MMSA population living below the 1999 U.S. Census Bureau-defined poverty threshold.

Among Hispanics that lived in low Hispanic segregation areas, almost 64% of Hispanic Blacks lived in areas characterized by a high non-Hispanic Black isolation score, compared with ≤ 32% for Hispanic Whites and Hispanics who identified as some other race (Table 3). In contrast, over 50% of all Hispanic Whites that lived in low Hispanic segregation areas lived in areas characterized by a high non-Hispanic White isolation score. This was true of just 20% of Hispanic Blacks and under 32% of Hispanics who identified as some other race.

**Table 3:** **Percentage of all Hispanic participants living in low Hispanic segregation areas**^**a **^**by level of non**-**Hispanic residential segregation and race**, **2003**-**2008 Behavioral Risk Factor Surveillance System** (**BRFSS**)

	**Hispanic blacks(n** **=** **319)**	**Hispanics who identified as some other race(n** **=** **1,469)**	**Hispanic whites(n** **=** **2,702)**
Quartile of non-hispanic black isolation index score			
NBI 1 (low)	9.1	43.5	41.0
NBI 2	2.5	5.0	5.6
NBI 3	24.8	21.0	21.4
NBI 4 (high)	63.6	30.5	32.0
Quartile of non-hispanic white isolation index score			
NWI 1 (low)	39.2	34.0	15.8
NWI 2	21.3	13.4	14.6
NWI 3	19.4	20.9	19.3
NWI 4 (high)	20.1	31.7	50.3

^a^Low Hispanic segregation areas is defined as the lowest quartile of the Hispanic isolation index.

*Abbreviation:NBI* = Non-Hispanic Black isolation index score; *NWI* = Non-Hispanic White isolation index score.

Among men, in both the minimally and fully adjusted model, segregation was unassociated with BMI (Table 4). There was no difference in mean BMI between Hispanic Black and White men, but Hispanic men who identified as some other race had higher mean BMI compared with white Hispanic men. Less acculturated men (as measured by language of interview) had lower mean BMI than more acculturated men, and lower educational attainment was associated with higher BMI. The relationship between segregation and BMI did not vary by race (P_interaction_ > 0.3).

**Table 4:** **Gender**-**stratified adjusted mean differences** (**standard error**) **in body mass index among Hispanics in the 2003**–**2008 Behavioral Risk Factor Surveillance System** (**BRFSS**)

	**Men (n** **=** **22,901)**		**Women (n** **=** **37,335)**	
	**Model 1**	**Model 2**	**Model 1**	**Model 2**
Hispanic isolation index	0.06 (0.12)	0.11 (0.13)	0.19 (0.11)*	0.07 (0.11)
Race				
Hispanic black	0.22 (0.26)	0.17 (0.26)	1.43 (0.31)**	1.39 (0.30)**
Hispanics who identified as some other race	0.26(0.10)**	0.29 (0.11)**	0.72 (0.11)**	0.55 (0.10)**
Hispanic white	ref	ref	ref	ref
Language of exam				
Non-English		−0.87 (0.12)*		−0.10 (0.14)
English		ref		ref
Education				
Less than high school		0.54 (0.14)**		2.19 (0.16)**
High school		0.57 (0.12)**		1.11 (0.10)**
More than high school		ref		ref

*P < 0.10; **P < 0.05.

All models are adjusted for age, age^2^, MMSA-level poverty, and MMSA population size.

*Abbreviation:MMSA* = metropolitan or micropolitan statistical area.

Among women, higher segregation was marginally associated with higher mean BMI (beta = 0.19 kg/m^2^; SE = 0.11) after adjusting for age, race, and MMSA-level confounders. However, this relationship weakened after controlling for language of interview and education (beta = 0.07; SE = 0.11). Both Hispanic Black and some other race women had higher mean BMI than Hispanic White women. In addition, lower educational attainment was associated with higher BMI.

**Figure 1:**
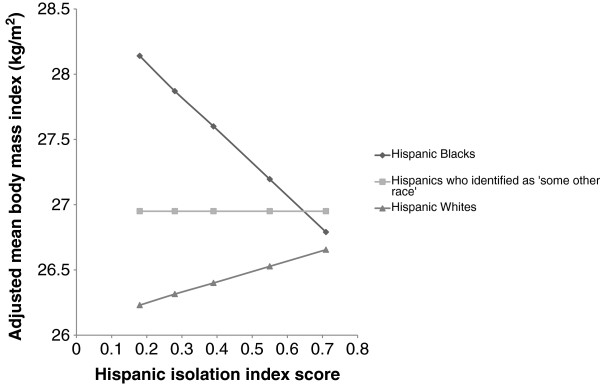
**Adjusted mean body mass index for Hispanic Black, White, and some other race women by level of the Hispanic isolation index.** Adjusted estimates calculated to correspond to the mean age of the sample (mean age = 46.2). Model was adjusted for age, age^2^, race, education, language of interview, MMSA population size, percentage of people in MMSA living below the U.S. Census Bureau-defined poverty threshold, Hispanic isolation index, and race-Hispanic isolation index interaction.

In contrast to findings among men, the relationship between Hispanic segregation and BMI varied by race among women (P_interaction_ for Hispanic Blacks = 0.03, P_interaction_ for Hispanics who identified as some other race = 0.09) (Figure 1). Higher segregation was associated with higher mean BMI for Hispanic White women (beta = 0.17 kg/m^2^; SE = 0.11; *P* = 0.11) but with lower BMI for Hispanic Black women (beta = −0.54 kg/m^2^; SE = 0.34; *P* = 0.12). Segregation was unassociated with mean BMI forHispanic women who identified as some other race (beta = −0.001 kg/m^2^; SE = 0.13; *P* = 0.99). Analogously, race differences in women were small in areas where Hispanic segregation was highest; but in areas of low segregation, there were considerable race disparities. To illustrate this we estimated associations of race with BMI at the lowest (0.24) and highest (0.54) quartiles of the Hispanic isolation index score. When segregation was low, Hispanic Black (beta = 1.71; SE = 0.28; *P* < 0.001) and some other race (beta = 0.67; SE = 0.14; *P* < 0.001) women had significantly higher mean BMI than Hispanic White women. When segregation was high, these differences were much smaller (beta = 0.70; SE = 0.49; *P* = 0.15 for Hispanic Black women and beta = 0.43; SE = 0.10; *P* < 0.001 for Hispanic women who identified as some other race).

## Discussion

We investigated the association between metropolitan-level ethnic residential segregation and BMI among Hispanics and whether this relationship varied by race. Hispanic segregation was unassociated with BMI among men, and there was no variation by race. Among women, the association between segregation and BMI was accounted for by acculturation and education, individual-level factors that may be on the causal pathway linking segregation to BMI. There was also significant heterogeneity in this relationship by race. Specifically, higher segregation was associated with higher mean BMI for Hispanic White women, but with lower mean BMI among Hispanic Black women, and there was no association among Hispanic women who identified as some other race. We also found that race differences in BMI were smaller for women living in areas of high Hispanic segregation than those living in low Hispanic segregation areas.

Few studies have examined the relationship between metropolitan-level residential segregation and obesity in Hispanics, and findings are mixed. A study of segregation and obesity among Hispanic adults using 2000 BRFSS data found segregation was associated higher obesity [20]. However, this study did not examine heterogeneity in this relationship by gender or race. Another study of metropolitan-level segregation and weight status in Hispanics was conducted in Mexican-Americans in the National Health and Nutrition Examination Survey [19]. Hispanic segregation was unassociated with obesity among men, but there was a negative relationship among women. These findings are consistent with the gendered nature of the results we present here, but they are also distinct with respect to the direction and significance of the association among women. Unfortunately a direct comparison of these two studies is difficult since BRFSS does not provide information about country of origin (and thus we cannot assess associations specifically for Mexican-Americans). Nevertheless, these contrasting findings in Mexican-American women compared to a single broad category of Hispanic women highlight the extent to which health effects of segregation may be expected to differ for different Hispanic subgroups. Given the considerable heterogeneity of the Hispanic population with respect to characteristics like nativity, immigration status, country of origin, and race [22,24], associations between segregation and health are likely dependent on such factors and on the associated opportunities afforded to various Hispanic groups as they assimilate in the U.S.

Our findings for Hispanic women lend some support to the segmented assimilation theory [18] and suggest race may have a persistent influence on segregation and its relationship with health among Hispanics. Previous research shows that, compared to Hispanic Blacks, Hispanic Whites are less segregated from non-Hispanic Whites and more segregated from non-Hispanic Blacks [29]. Thus, Hispanic Whites living in low Hispanic segregation areas may be more likely to reside in predominantly non-Hispanic White neighborhoods. Living in these neighborhoods may be associated with lower BMI through lower levels of concentrated poverty and closer proximity to supermarkets [41,42]. In contrast, Hispanic Blacks living in low Hispanic segregation areas may be more likely to reside in poor quality, predominantly non-Hispanic Black neighborhoods as a result of race-related forces such as housing discrimination and neighbors’ hostility [29]. Residence in such areas may be associated with a higher BMI as a result of poorer quality and limited availability of health-promoting social and physical environmental resources (e.g., limited healthy food availability and lower neighborhood safety) [6,41,43]. This explanation is supported by our descriptive findings that Hispanic Blacks living in low Hispanic segregation areas were more likely to live in high non-Hispanic Black segregation areas, while Hispanic Whites living in low Hispanic segregation areas were more likely to live in high non-Hispanic White segregation areas. Unfortunately, because we do not have neighborhood-level information, we cannot ascertain the racial or ethnic composition of the actual neighborhoods where Hispanic Blacks and Whites are residing within these different MMSAs.

In addition to the differential residential opportunities available to Hispanic Blacks and Whites living in low Hispanic segregation areas, living in predominantly Hispanic neighborhoods may also have different health implications for Hispanic Blacks and Whites. For Hispanic Blacks, living in predominantly Hispanic neighborhoods may be associated with lower BMI by protecting them from the stress of exposure to discrimination [44,45] and providing them with better access to healthy foods [14,17]. This is consistent with the ethnic density hypothesis which posits that for minorities, living in communities with a high density of one’s own race or ethnic group may be health-promoting due to the presence of strong social networks and reduced exposure to direct prejudice [46]. This is also supported by several studies among Hispanics (largely Mexican Americans) that have shown that living in a predominantly Hispanic neighborhood is associated with better health including birth outcomes, mortality, and self-rated health [47].

Another key finding of this paper is that race differences in BMI were substantially smaller for those living in highly segregated Hispanic areas than those in less segregated areas. This is similar to findings from other studies that examined race differences among non-Hispanics living in similar environmental conditions. For example, non-Hispanic Blacks and Whites living in an integrated community in Baltimore resemble each other more closely in terms of obesity, diabetes, and hypertension prevalence than do non-Hispanic Blacks and Whites in national samples [2,48,49]. In addition, a previous study on metropolitan-level racial segregation and hypertension showed Black-White differences in hypertension prevalence was smaller for those living in low Black segregation, higher poverty neighborhoods [12]. One notable difference between our findings for Hispanics and these previous studies is that the smaller race differences among non-Hispanics were the result of prevalence among Whites being higher in those more integrated communities, not because prevalence was also lower for Blacks. Nevertheless, these findings underscore the role that contextual factors may play in the emergence of race disparities, even among Hispanics.

It is not clear why segregation was unrelated to BMI among Hispanic women who identified as some other race. The null association may be a reflection of the heterogeneity within that group, both in terms of the reasons that they may self-identify as some other race, and in terms of how they are perceived and treated by others (including other actors in housing markets). Research suggests that with increased assimilation, Hispanics who identified as some other race are less segregated from non-Hispanic Whites than Hispanic Blacks [29]. Therefore, Hispanics who identified as some other race may not experience the same level of housing discrimination, and may have more opportunities for upward mobility and assimilation than Hispanic Blacks, but perhaps less so than Hispanic Whites. Further research is needed in datasets with more detailed information on nativity and country of origin to better understand this finding.

This study is not without limitations. One is that BMI was calculated using self-reported height and weight. Studies show that BMI based on self-report tends to be under-reported [50]. However, there is no evidence of systematic differences in measurement error by race [51]. BRFSS is one of the few studies with sufficient numbers of Hispanic Blacks, which makes using this dataset a strength that outweighs the potential limitations of using self-reported data. In addition, the large sample size increases the likelihood that the overall patterns of association were still captured despite possible underreporting of BMI. Another limitation is that we did not have neighborhood-level information on participants and only had limited individual-level health behavioral data across the BRFSS survey years. Thus, we were unable to explore the neighborhood- and individual-level pathways through which residential segregation at the metropolitan level may impact BMI.

We also could not distinguish between Hispanic BRFSS participants that were foreign-born or U.S.-born. Previous research suggests that the association between residence in ethnic enclaves and health varies by nativity [21]. The difference in associations by race for women may have been confounded if the immigrant composition for Hispanic Blacks and Whites differed. In the 2000 U.S. Census, Hispanic Blacks were less likely to be foreign-born than Hispanics of other races [52]. We attempted to address this issue by including ‘language of interview’ as a covariate, which is a commonly used marker of assimilation [36], even though it may not be perfectly correlated with nativity (i.e. not all immigrants are unable to speak English). Nevertheless, future work in this area may benefit from the inclusion of additional measures of assimilation.

Finally, this study is limited by its cross-sectional design. Factors associated with obesity (e.g. health problems due to obesity or desire to live in areas that promote physical activity) may influence selection into metropolitan areas. Although we cannot rule out this selection bias, it is a smaller threat to validity in metropolitan-level segregation studies than in neighborhood-level segregation studies [53]. In addition, residential segregation is hypothesized to act on health across the life course [7]; assessing this relationship at one point in time might not accurately capture the true impact of this process on health.

## Conclusions

This is among the first studies to assess the association between metropolitan-level ethnic residential segregation and BMI among Hispanics and to examine heterogeneity by race. Our findings demonstrated racial variation in the association between segregation and mean BMI among Hispanic women. This heterogeneity points to the persistence of race in the U.S. as a driver of structural processes that may limit exposure to health-promoting aspects of residential environments. It also highlights the multiple forms of assimilation that exist among Hispanics and the implications of these assimilation processes for health.

Future work will need to assess the extent that housing discrimination and other forms of blocked mobility limits opportunities for Hispanics in urban areas. Effective solutions to health disparities will need to target the processes that create segregation and the differential conditions under which individuals live. Better characterization of the roles of racial categorization and related individual- and area-level factors as contributors to variation in disease among Hispanics may facilitate the design of more comprehensive solutions to prevent adverse health outcomes within the fastest growing demographic group in the U.S.

## Abbreviations

BMI: Body mass index; MMSA: Metropolitan or micropolitan statistical area; BRFSS: Behavioral risk factor surveillance system.

## Competing interests

The authors have no competing interests to declare.

## Authors’ contributions

Both authors conceived of the study and drafted the manuscript. KNK conducted the analyses. Both authors read and approved the final manuscript.

## Pre-publication history

The pre-publication history for this paper can be accessed here:

http://www.biomedcentral.com/1471-2458/14/283/prepub

## References

[B1] Humes KR Jones NA Ramirez RR Overview of Race and Hispanic Origin: 2010 Census Briefs 2011 U.S. Census Bureau: Washington, D. C

[B2] Bleich SN Thorpe RJ Jr Sharif-Harris H Fesahazion R Laveist TA Social context explains race disparities in obesity among women J Epidemiol Community Health 2010 64 465 469 10.1136/jech.2009.096297 20445215 PMC3099623

[B3] Burdette HL Wadden TA Whitaker RC Neighborhood safety, collective efficacy, and obesity in women with young children Obesity (Silver Spring) 2006 14 518 525 10.1038/oby.2006.67 16648624

[B4] Dubowitz T Ghosh-Dastidar M Eibner C Slaughter ME Fernandes M Whitsel EA Bird CE Jewell A Margolis KL Li W Michael YL Shih RA Manson JE Escarce JJ The Women’s Health Initiative: the food environment, neighborhood socioeconomic status, BMI, and blood pressure Obesity (Silver Spring) 2012 20 862 871 10.1038/oby.2011.141 21660076 PMC4018819

[B5] Inagami S Cohen DA Brown A FAsch SM Body mass index, neighborhood fast food and restaurant concentration, and car ownership J Urban Health 2009 86 683 695 10.1007/s11524-009-9379-y 19533365 PMC2729867

[B6] Mujahid MS Roux AV Shen M Gowda D Sanchez B Shea S Jacobs DR Jackson SA Jr Relation between neighborhood environments and obesity in the Multi-Ethnic Study of Atherosclerosis Am J Epidemiol 2008 167 1349 1357 10.1093/aje/kwn047 18367469

[B7] Williams DR Collins C Racial residential segregation: a fundamental cause of racial disparities in health Public Health Rep 2001 116 404 416 10.1016/S0033-3549(04)50068-7 12042604 PMC1497358

[B8] Fischer MJ Tienda M Tienda M, Mitchell F Redrawing Spatial Color Lines: Hispanic Metropolitan Dispersal, Segregation, and Economic Opportunity Hispanics and the Future of America 2006 Washington, D.C: The National Academies Press 100 137 20669436

[B9] Massey DS Denton NA American Apartheid: Segregation and the Making of the Underclass 1993 Cambridge, MA: Harvard University Press

[B10] Chang VW Racial residential segregation and weight status among US adults Soc Sci Med 2006 63 1289 1303 10.1016/j.socscimed.2006.03.049 16707199

[B11] Corral I Landrine H Hao Y Zhao L Mellerson JL Cooper DL Residential segregation, health behavior and overweight/obesity among a national sample of African American adults J Health Psychol 2012 17 371 378 10.1177/1359105311417191 21844135

[B12] Kershaw KN Diez Roux AV Burgard SA Lisabeth LD Mujahid MS Schulz AJ Metropolitan-level racial residential segregation and black-white disparities in hypertension Am J Epidemiol 2011 Published online ahead of print June 24, 2011: (doi:10.1093/aje/kwr1116) 10.1093/aje/kwr116 PMC3202148 21697256

[B13] Subramanian SV Acevedo-Garcia D Osypuk TL Racial residential segregation and geographic heterogeneity in black/white disparity in poor self-rated health in the US: a multilevel statistical analysis Soc Sci Med 2005 60 1667 1679 10.1016/j.socscimed.2004.08.040 15686800

[B14] Osypuk TL Diez Roux AV Hadley C Kandula NR Are immigrant enclaves healthy places to live? The Multi-ethnic Study of Atherosclerosis Soc Sci Med 2009 69 110 120 10.1016/j.socscimed.2009.04.010 19427731 PMC2750873

[B15] Galster G Metzger K Waite R Neighborhood opportunity structures of immigrant populations, 1980 and 1990 Housing Policy Debate 1999 10 395 442 10.1080/10511482.1999.9521337

[B16] Fernandez Kelly MP Shauffler R Portes A Divided fates: immigrant children and the new assimilation The new second generation 1996 New York, NY: Russell Sage Foundation 30 53

[B17] Park Y Neckerman K Quinn J Weiss C Jacobson J Rundle A Neighbourhood immigrant acculturation and diet among Hispanic female residents of New York City Public Health Nutr 2011 14 1593 1600 10.1017/S136898001100019X 21414245 PMC3696981

[B18] Portes A Zhou M The new second generation: Segmented assimilation and its variants among post-1965 immigrant youth Ann Am Acad Pol Soc Sci 1993 530 74 96 10.1177/0002716293530001006

[B19] Kershaw KN Albrecht SS Carnethon MR Racial and ethnic residential segregation, the neighborhood socioeconomic environment, and obesity among Blacks and Mexican Americans Am J Epidemiol 2013 177 299 309 10.1093/aje/kws372 23337312 PMC3566709

[B20] Corral I Landrine H Zhao L Residential segregation and obesity among a national sample of Hispanic adults J Health Psychol 2013 19 503 508 23460679 10.1177/1359105312474912

[B21] Osypuk TL Bates LM Acevedo-Garcia D Another Mexican birthweight paradox? The role of residential enclaves and neighborhood poverty in the birth weight of Mexican-origin infants Soc Sci Med 2010 70 550 560 10.1016/j.socscimed.2009.10.034 19926186 PMC2815074

[B22] Grieco EM Cassidy RC Overview of race and Hispanic origin: Census brief 2000 Washington, DC: U.S. Cenus Bureau

[B23] Logan JR Jimenez Roman M, Flores J How race counts for Hispanic Americans The Afro-Latin@ Reader: History and Culture in the United States 2010 Durham, NC: Duke University Press

[B24] Taylor P Lopez MH Martínez JH Velasco G When labels don’t fit: hispanics and their views of identity 2012 Washington, DC: Pew Hispanic Center

[B25] Lopez N Gomez LE, Lopez N Contextualizing lived race-gender and the racialized-gendered social determinants of health Mapping “race”: critical approaches to health disparities research 2013 New Brunswick, NJ: Rutgers University Press 179 211

[B26] Gravlee CC Dressler WW Bernard HR Skin color, social classification, and blood pressure in southeastern Puerto Rico Am J Public Health 2005 95 2191 2197 10.2105/AJPH.2005.065615 16257938 PMC1449506

[B27] Jones CP Truman BI Elam-Evans LD Jones CA Jones CY Jiles R Rumisha SF Perry GS Using “socially assigned race” to probe white advantages in health status Ethn Dis 2008 18 496 504 19157256

[B28] Glazer N Moynihan DP Beyond the Melting Pot: The Negroes, Puerto Ricans, Jews, Italians, and Irish of New York City 1970 2 Cambridge, MA: MIT Press

[B29] Iceland J Nelson KA Hispanic segregation in metropolitan America: exploring the multiple forms of spatial assimilation Am Sociol Rev 2008 73 741 765 10.1177/000312240807300503

[B30] Alba R Nee V Remaking the American Mainstream 2003 Cambridge, MA: Harvard University Press

[B31] Massey DS Ethnic residential segregation: a theoretical synthesis and empirical review Sociol Soc Res 1985 69 315 350

[B32] Centers for Disease Control and Prevention Behavioral Risk Factor Surveillance System Survey Data 2003-2008 Atlanta, GA: Centers for Disease Control and Prevention

[B33] Massey DS Denton NA The dimensions of residential segregation Soc Forces 1988 67 281 315 10.1093/sf/67.2.281

[B34] US Census Bureau Design and Methodology, American Community Survey 2009 Washington, DC: U.S. Government Printing Office

[B35] Acevedo-Garcia D Lochner KA Osypuk TL Subramanian SV Future directions in residential segregation and health research: a multilevel approach Am J Public Health 2003 93 215 221 10.2105/AJPH.93.2.215 12554572 PMC1447719

[B36] Lara M Gamboa C Kahramanian MI Morales LS Bautista DE Acculturation and Latino health in the United States: a review of the literature and its sociopolitical context Annu Rev Public Health 2005 26 367 397 10.1146/annurev.publhealth.26.021304.144615 15760294 PMC5920562

[B37] Bureau of the Census Current Population Survey, 1999 1999 Washington, D.C.: Bureau of the Census

[B38] Block JP He Y Zaslavsky AM Ding L Ayanian JZ Psychosocial stress and change in weight among US adults Am J Epidemiol 2009 170 181 192 10.1093/aje/kwp104 19465744 PMC2727271

[B39] Grunberg NE Straub RO The role of gender and taste class in the effects of stress on eating Health Psychol 1992 11 97 100 1582385 10.1037//0278-6133.11.2.97

[B40] Carle AC Fitting multilevel models in complex survey data with design weights: Recommendations BMC Med Res Methodol 2009 9 49 10.1186/1471-2288-9-49 19602263 PMC2717116

[B41] Moore LV Diez Roux AV Associations of neighborhood characteristics with the location and type of food stores Am J Public Health 2006 96 325 331 10.2105/AJPH.2004.058040 16380567 PMC1470485

[B42] Zenk SN Schulz AJ Israel BA James SA Bao S Wilson ML Neighborhood racial composition, neighborhood poverty, and the spatial accessibility of supermarkets in metropolitan Detroit Am J Public Health 2005 95 660 667 10.2105/AJPH.2004.042150 15798127 PMC1449238

[B43] Mujahid MS Diez Roux AV Cooper RC Shea S Williams DR Neighborhood stressors and race/ethnic differences in hypertension prevalence (the Multi-Ethnic Study of Atherosclerosis) Am J Hypertens 2011 24 187 193 10.1038/ajh.2010.200 20847728 PMC3319083

[B44] Hunte HE Association between perceived interpersonal everyday discrimination and waist circumference over a 9-year period in the Midlife Development in the United States cohort study Am J Epidemiol 2011 173 1232 1239 10.1093/aje/kwq463 21354988 PMC3139961

[B45] Hunte HE Williams DR The association between perceived discrimination and obesity in a population-based multiracial and multiethnic adult sample Am J Public Health 2009 99 1285 1292 10.2105/AJPH.2007.128090 18923119 PMC2696650

[B46] Halpern D Minorities and mental health Soc Sci Med 1993 36 597 607 10.1016/0277-9536(93)90056-A 8456329

[B47] Becares L Shaw R Nazroo J Stafford M Albor C Atkin K Kiernan K Wilkinson R Pickett K Ethnic density effects on physical morbidity, mortality, and health behaviors: a systematic review of the literature Am J Public Health 2012 102 e33 e66 23078507 10.2105/AJPH.2012.300832 PMC3519331

[B48] Laveist TA Thorpe RJ Jr Galarraga JE Bower KM Gary-Webb TL Environmental and socio-economic factors as contributors to racial disparities in diabetes prevalence J Gen Intern Med 2009 24 1144 1148 10.1007/s11606-009-1085-7 19685264 PMC2762509

[B49] Thorpe RJ Jr Brandon DT Laveist TA Social context as an explanation for race disparities in hypertension: findings from the Exploring Health Disparities in Integrated Communities (EHDIC) Study Soc Sci Med 2008 67 1604 1611 10.1016/j.socscimed.2008.07.002 18701200 PMC3521570

[B50] Gorber SC Tremblay M Moher D Gorber B A comparison of direct vs. self-report measures for assessing height, weight and body mass index: a systematic review Obes Rev 2007 8 307 326 10.1111/j.1467-789X.2007.00347.x 17578381

[B51] Ezzati M Martin H Skjold S Vander Hoorn S Murray CJ Trends in national and state-level obesity in the USA after correction for self-report bias: analysis of health surveys J R Soc Med 2006 99 250 257 10.1258/jrsm.99.5.250 16672759 PMC1457748

[B52] Ruggles S Alexander JT Genadek K Goeken R Schroeder MB Sobek M Integrated public use microdata series: Version 5.0 [Machine-readable database] 2010 Minneapolis: University of Minnesota

[B53] Cutler DM Glaeser EL Are ghettos good or bad? Q J Econ 1997 112 827 872 10.1162/003355397555361

